# Transcriptome sample statistics for the sugar beet root maggot (*Tetanops myopaeformis*) infecting sugar beet

**DOI:** 10.6026/9732063002001881

**Published:** 2024-12-31

**Authors:** Sudha Acharya, Nadim W. Alkharouf, Muhammad Massub Tehseen, Chenggen Chu, Vincent P. Klink

**Affiliations:** 1 Department of Computer and Information Sciences, Towson University, Towson, MD, 21252, USA; 2 USDA-ARS-NA, Northern Great Plains Research Laboratory, 1307 N 18TH ST Northern Crop Science Laboratory, Fargo, ND 58102, USA; 3 Department of Plant Sciences, North Dakota State University, Fargo, ND 58102, USA; 4 USDA-ARS-NEA-BARC, Molecular Plant Pathology Laboratory, Building 004, Room 122, BARC- West, 10300 Baltimore Ave., Beltsville, MD 20705, USA

**Keywords:** Sugar beet root maggot, *Tetanops myopaeformis*, sugar beet, *Beta vulgaris*, transcriptome, resistant, susceptible, RNA-seq

## Abstract

The sugar beet root maggot (SBRM), *Tetanops myopaeformis* (von Röder) insect pathogen devastates sugar beet (SB), *Beta vulgaris* ssp, vulgaris (*B. vulgaris*), one of only two plants from which significant global raw sugar is produced, $1B, U.S., $4.6 B, globally. Larval SBRMs experiencing F1010 and L19 susceptible or F1016 and F1024 resistant SB responses are RNA sequenced, sampled at time = 0 hours post infection [hpi], 24, 48 and 72 hpi. Transcriptomic analyses determined the number of reads per sample, mapped the transcripts to the recently sequenced SBRM TmSBRM_v1.0 draft genome and identified genes that relate to the resistant and susceptible responses. The RNA-seq study provides data for generating differential expression analyses, yielding an understanding SBRM biology, control strategy development, relationship to model and non-model organisms and aiding sugar beet improvement for stakeholders.

## Background:

The fully referenced version of this work is available [1]. *Beta vulgaris* ssp, vulgaris (*B. vulgaris*), sugar beet (SB), Order Carophyllales, Family Amaranthaceae, is one of two plants, globally, from which sugar is widely produced with a worldwide value of $4.6 B and $1 B U.S., harvested from 1.14 million acres of land [2]. Upon U.S. introduction, SB was encountered by the native insect pathogen *T. myopaeformis* (SBRM) on which it can complete its life cycle and while it can complete its life cycle on other non-native plant species, the native SBRM host has not yet been identified [3, 4]. SBRM is the most devastating SB pathogen in North America where it can decrease yield by up to 100%, locally and of further concern is its increasing geographic spread [1, 5, 6, 7, 8, 9, 10, 11, 12, 13, 14, 15, 16, 17-18]. Transcriptomic knowledge has facilitated the pathogenic nature of other insects [1]. In the presented analysis, larval SBRMs experiencing F1010 and L19 susceptible or F1016 and F1024 resistant SB responses are RNA sequenced, sampled at time = 0, 24, 48 and 72 hpi, for scientific study of its pathogenicity and stakeholder benefit [19].

## Materials and Methods:

## Plant infection:

SBRM larvae were collected in mid-June 2022 from a field location close to St. Thomas, ND. After cleaning all larvae using 1% Clorox Germicidal Bleach, the 1- and 2-instar larvae were used for root infestation of *B. vulgaris* F1016 (PI 608437) and F1024 (PI 658654) that are resistant and F1010 (PI 535818) and L19 (PI 590690) that are susceptible genotypes [1, 20, 21-22]. The infestation experiment included three replications for each genotype with three plants infested in each replication. For preparing roots for infestation, seeds were germinated using 1% hydrogen peroxide solution [23] and germinated seeds were planted in a greenhouse room under 16:8 (day: night) light regime with temperature range between 20-30°C. Roots were collected 4 weeks after planting. After being cleaned to remove the soil, three roots of each genotype as one replication were placed on a 15 cm x 10 cm, 0.8% agar plate [24]. Subsequently, fifteen 1- or 2-instar larvae were added to each plate with 5 larvae per root. All plates were then kept in dark at 28°C. Root and insect samples were collected at 0 hpi (right before infestation) and subsequently at 24, 48 and 72 hpi. All samples were immediately flash frozen into liquid nitrogen and then stored at -80°C before RNA isolation and subsequent RNA-seq data generation.

## RNA isolation:

Flash-frozen SBRM larval samples were sent to Omega Bioservices Inc., 400 Pinnacle Way, Ste 425, Norcross, GA 30071 for RNA isolation, quality assurance and RNA sequencing according to Alsherhi *et al.* [25]. In brief, the RNA isolation implemented a well-established protocol for RNA isolation and library preparation to achieve high-quality sequencing data. The Omega Biotek E.Z.N.A. ® Total RNA Kit (Omega Bio-tek) was used to extract total RNA from the samples, following the manufacturer's protocol. The concentration and integrity of the RNA were assessed using a Nanodrop 2000c spectrophotometer (Thermo Scientific Inc.) and an Agilent 4150 TapeStation instrument (Agilent Technologies), respectively.

## RNA library preparation:

For library generation, up to 1 mg of total RNA was used according to the manufacturer's instructions for the NEBNext® Poly(A) mRNA Magnetic Isolation Module E7490L and NEBNext® Ultra^TM^ II Directional RNA Library Prep Kit for Illumina® E7760L (New England Biolabs Inc.). Quality and quantity evaluation of the libraries were conducted using the High Sensitivity D1000 Screen Tape on an Agilent 4150 TapeStation instrument. Subsequently, the libraries underwent normalization, pooling and were sequenced with Illumina Novaseq X Plus instrument (Illumina, Inc.) following the manufacturer's recommendations.

## RNA-seq data processing:

For the study presented here, the RNA-seq data analysis process used Geneious prime (https://www.geneious.com/), version 2024.0 with the steps of that pipeline detailed at https://www.geneious.com/series/expression-analysis. The analysis process presented here involved sequence trimming, alignment and counting. Trimming was used to increase the read's mapping rate by eliminating adapter sequences and removing poor-quality nucleotides. The alignment was performed to the SBRM TmSBRM_v1.0 draft genome. After mapping the reads, they were assigned to a gene or transcript in a process known as counting or quantification. This step was followed by a normalization procedure employed to remove possible sequencing bias.

## Results:

## RNA-seq data processing:

**Figure 1:**
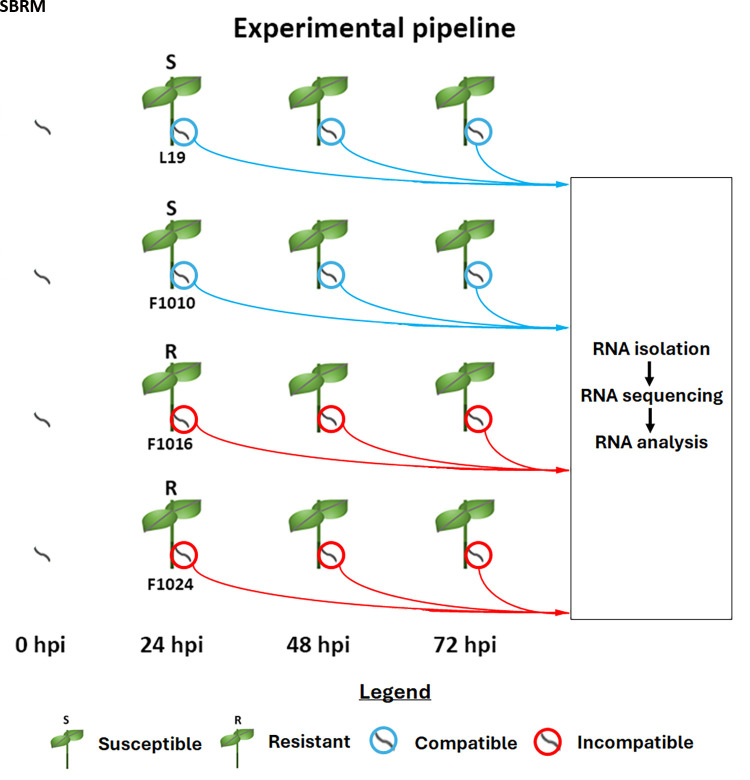
Experimental Pipeline

The SBRM-susceptible L19 and F1010 and SBRM-resistant F1016 and F1024 *B. vulgaris* genotypes have been obtained. The genotypes were used in experiments that isolated SBRM larval RNA. The SBRM larval RNA was used for RNA seq experiments. The experimental pipeline is presented (Figure 1). The RNA seq sample statistics are presented (Table 1).

The SBRM-susceptible L19 and F1010 and SBRM-resistant F1016 and F1024 *B. vulgaris* genotypes are shown. The respective compatible and incompatible SBRM are encircled by a blue or red ring. At t = 0 hpi, the SBRM were collected before any introduction to *B. vulgaris*. Thus, the SBRM are shown to not be closely associated with *B. vulgaris*. SBRM are subsequently shown to be in direct contact with *B. vulgaris* at the t = 24, 48 and 72 hpi time points. The samples were collected for transcriptomic study that involved RNA isolation, sequencing and analysis. The experimental pipeline is presented (Figure 1). The RNA-seq analysis has resulted in acquiring data for each of the 39 samples (Table 1). The reads have then been mapped to the recently sequenced SBRM TmSBRM_v1.0 draft genome. This analysis has allowed for the generation of a general assessment of gene activity on the SBRM TmSBRM_v1.0 draft genome, aided by its annotation.

## Discussion:

The RNA-seq analysis has identified a range in total processed reads per sample of 37,947,020 to 46,319,624 in total assembled (used) reads per sample of 25,883,749 (66.16%) to 29,385,047 (70.67%) and a range in total unassembled reads per sample of 10,538,861 (26.64%) to 12,303,540 (29.07%). The range in average used reads per time point was 23,246 (L19 resistant, 24 hpi) to 24,525 (F1010 resistant, 72 hpi), 5.22% therefore, the sample read quantity is similar between the different samples. From these data, further processing is possible, with the advancement of science being that the research allows for an idea of differential expression of genes during the susceptible and resistant reactions, the identification of genes, gene pathways and biological processes which may or may not fall under gene pathways to be identified, scientists to devise management, control and biological assays for SBRM much in the same way that has been done for other devastating agricultural pathogens [26, 27- 28]. A preprint outlining the framework of this manuscript and more details relating to the introduction are presented [1].

## Conclusion:

Transcriptomes have been generated for larval SBRMs experiencing F1010 and L19 susceptible or F1016 and F1024 resistant SB responses, sampled at time = 0 hours post infection [hpi], 24, 48 and 72 hpi. RNA sequences are identified that map or do not map to the reference genome. The sequences are a resource to understand SBRM biology during susceptible and resistant reactions for stakeholder benefit.

## Ethics statement:

The authors have read and follow the ethical requirements for publication in Bioinformation and confirming that the current work does not involve human subjects, animal experiments, or any data collected from social media platforms.

## Author credit statement:

[1] SA has been involved in Methodology; Software; Validation; Formal analysis; Investigation; Resources; Data Curation; Writing - Original Draft.

[2] NA has been involved in Methodology; Software; Validation; Formal analysis; Investigation; Resources; Data Curation; Writing - Original Draft.

[3] MT has been involved in Investigation; Resources.

[4] CC has been involved in Investigation; Resources, Supervision; Project administration; Funding acquisition.

[5] VK has been involved in Conceptualization; Methodology; Resources; Visualization; Supervision; Project administration; Funding acquisition; Writing - Original Draft.

## Declaration of competing interests:

The authors declare that they have no known competing financial interests or personal relationships that could have appeared to influence the work reported in this paper.

## Figures and Tables

**Table 1:** Transcriptome statistics

**Sample NO.**	**SBRM use**	**SB genotype**	**Outcome**	**Time point**	**Total processed reads**	**Assembled (Used reads)**	**% Assembled**	**Unassembled**	**% Unassembled**
1	SBRM control	no SB*	n/a	0 hpi	41,366,384	29,614,682	71.59117896	11,751,702	28.40882104
2	SBRM control	no SB*	n/a	0 hpi	45,730,450	32,709,379	71.52647525	13,021,071	28.47352475
3	SBRM control	no SB*	n/a	0 hpi	38,753,604	27,516,689	71.00420647	11,236,915	28.99579353
4	SBRM infested	F1024	resistant	24 hpi	44,942,632	32,898,251	73.20054375	12,044,381	26.79945625
5	SBRM infested	F1024	resistant	24 hpi	43,740,534	31,705,744	72.48595548	12,034,790	27.51404452
6	SBRM infested	F1024	resistant	24 hpi	41,578,980	29,385,047	70.67284238	12,193,933	29.32715762
7	SBRM infested	F1016	resistant	24 hpi	42,906,878	30,892,119	71.99805821	12,014,759	28.00194179
8	SBRM infested	F1016	resistant	24 hpi	45,633,030	33,527,362	73.47169802	12,105,668	26.52830198
9	SBRM infested	F1016	resistant	24 hpi	37,947,020	27,188,332	71.64813469	10,758,688	28.35186531
10	SBRM infested	F1010	susceptible	24 hpi	45,366,574	32,685,868	72.04834996	12,680,706	27.95165004
11	SBRM infested	F1010	susceptible	24 hpi	39,166,906	27,856,471	71.12247008	11,310,435	28.87752992
12	SBRM infested	F1010	susceptible	24 hpi	39,703,208	28,549,398	71.90703079	11,153,810	28.09296921
13	SBRM infested	L19	susceptible	24 hpi	38,527,460	27,743,031	72.00846098	10,784,429	27.99153902
14	SBRM infested	L19	susceptible	24 hpi	39,363,640	28,339,559	71.99425409	11,024,081	28.00574591
15	SBRM infested	L19	susceptible	24 hpi	46,319,624	33,639,849	72.62547943	12,679,775	27.37452057
16	SBRM infested	F1024	resistant	48 hpi	41,549,788	28,818,653	69.35932621	12,731,135	30.64067379
17	SBRM infested	F1024	resistant	48 hpi	40,742,024	28,661,525	70.34880005	12,080,499	29.65119995
18	SBRM infested	F1024	resistant	48 hpi	45,219,784	32,076,880	70.93550027	13,142,904	29.06449973
19	SBRM infested	F1016	resistant	48 hpi	38,964,178	27,309,685	70.08921117	11,654,493	29.91078883
20	SBRM infested	F1016	resistant	48 hpi	40,442,336	29,203,809	72.21098455	11,238,527	27.78901545
21	SBRM infested	F1016	resistant	48 hpi	39,566,764	29,027,903	73.36435954	10,538,861	26.63564046
22	SBRM infested	F1010	susceptible	48 hpi	42,317,332	30,013,792	70.92552999	12,303,540	29.07447001
23	SBRM infested	F1010	susceptible	48 hpi	43,407,312	30,678,669	70.67626993	12,728,643	29.32373007
24	SBRM infested	F1010	susceptible	48 hpi	42,600,156	29,429,372	69.08277988	13,170,784	30.91722012
25	SBRM infested	L19	susceptible	48 hpi	41,129,322	29,218,917	71.04157224	11,910,405	28.95842776
26	SBRM infested	L19	susceptible	48 hpi	38,589,096	27,004,810	69.98041623	11,584,286	30.01958377
27	SBRM infested	L19	susceptible	48 hpi	41,630,316	29,920,707	71.87239943	11,709,609	28.12760057
28	SBRM infested	F1024	resistant	72 hpi	43,111,724	29,379,202	68.1466647	13,732,522	31.8533353
29	SBRM infested	F1024	resistant	72 hpi	41,201,980	28,859,219	70.0432819	12,342,761	29.9567181
30	SBRM infested	F1024	resistant	72 hpi	39,162,290	27,308,993	69.73288079	11,853,297	30.26711921
31	SBRM infested	F1016	resistant	72 hpi	40,314,630	28,519,330	70.741887	11,795,300	29.258113
32	SBRM infested	F1016	resistant	72 hpi	42,578,254	29,420,002	69.09630912	13,158,252	30.90369088
33	SBRM infested	F1016	resistant	72 hpi	42,440,440	28,561,396	67.29759635	13,879,044	32.70240365
34	SBRM infested	F1010	susceptible	72 hpi	44,288,560	29,460,791	66.52009232	14,827,769	33.47990768
35	SBRM infested	F1010	susceptible	72 hpi	44,569,173	29,849,449	66.97330686	14,719,724	33.02669314
36	SBRM infested	F1010	susceptible	72 hpi	39,120,669	25,883,749	66.16387107	13,236,920	33.83612893
37	SBRM infested	L19	susceptible	72 hpi	40,681,422	28,238,881	69.41468516	12,442,541	30.58531484
38	SBRM infested	L19	susceptible	72 hpi	41,919,080	28,609,391	68.24909087	13,309,689	31.75090913
39	SBRM infested	L19	susceptible	72 hpi	38,559,366	27,700,699	71.83909352	10,858,667	28.16090648
